# Quality indicators in headache care: an implementation study in six Italian specialist-care centres

**DOI:** 10.1186/s10194-017-0762-x

**Published:** 2017-05-05

**Authors:** L. Pellesi, S. Benemei, V. Favoni, C. Lupi, E. Mampreso, A. Negro, M. Paolucci, T. J. Steiner, M. Ulivi, S. Cevoli, S. Guerzoni

**Affiliations:** 1 Medical Toxicology - Headache Centre, Policlinic Hospital, University of Modena and Reggio Emilia, Modena, Italy; 2 Department of Health Sciences, Section of Clinical Pharmacology and Oncology, University of Florence, Florence, Italy; 3 IRCCS Institute of Neurological Sciences of Bologna, Department of Biomedical and Neuromotor Sciences DIBINEM, University of Bologna, Bologna, Italy; 4 Department of Neurosciences, Headache Centre, University of Padua, Padua, Italy; 5 Department of Clinical and Molecular Medicine, Sapienza University of Rome, Sant’Andrea Hospital, Rome, Italy; 6 Headache Centre, Neurology Unit, University Campus Bio-Medico, Rome, Italy; 7 Department of Neuromedicine and Movement Science, NTNU Norwegian University of Science and Technology, Trondheim, Norway; 8 Division of Brain Sciences, Imperial College London, London, UK; 9 IRCCS Institute of Neurological Sciences of Bologna, Bologna, Italy

**Keywords:** Headache disorders, Headache care, Service quality evaluation, Benchmarking, Global Campaign against Headache

## Abstract

**Background:**

Headache disorders are highly prevalent, and have a substantial and negative impact on health worldwide. They are largely treatable, but differences in structure, objectives, organization and delivery affect the quality of headache care. In order to recognize and remedy deficiencies in care, the Global Campaign against Headache, in collaboration with the European Headache Federation, recently developed a set of quality indicators for headache services. These require further assessment to demonstrate fitness for purpose. This is their first implementation to evaluate quality in headache care as a multicentre national study.

**Methods:**

Between September and December 2016, we applied the quality indicators in six Italian specialist headache centres (Bologna, Firenze, Modena, Padova, Roma Campus Bio-Medico and Roma Sapienza). We used five previously developed assessment instruments, translated into Italian according to *Lifting The Burden’s* translation protocol for hybrid documents. We took data from 360 consecutive patients (60 per centre) by questionnaire and from their medical records, and by different questionnaires from their health-care providers (HCPs), including physicians, nurses, psychologists and nursing assistants.

**Results:**

The findings, comparable between centres, confirmed the feasibility and practicability of using the quality indicators in Italian specialist headache centres. The questionnaires were easily understood by HCPs and patients, and were not unduly time-consuming. Diagnoses were almost all (> 97%) according to ICHD criteria, and routinely (100%) reviewed during follow-up. Diagnostic diaries were regularly used by 96% of physicians. Referral pathways from primary to specialist care existed in five of the six clinics, as did urgent referral pathways. Instruments to assess disability and quality of life were not used regularly, a deficiency that needs to be addressed.

**Conclusion:**

This Italy-wide survey confirmed in six specialist centres that the headache service quality indicators are fit for purpose. By establishing majority practice, identifying commonalities and detecting deficits as a guide to quality improvement, the quality indicators may be used to set benchmarks for quality assessment. The next step is extend use and evaluation of the indicators into non-specialist care.

## Background

Headache disorders are highly prevalent, with major negative impacts on health, well-being and productivity worldwide. They are treatable, but differences in structure, objectives, organization and delivery affect the quality of medical care provided for them. While service quality is one of the most important factors influencing outcomes delivered by a health system, “quality” does not have clear meaning in this context, and its measurement is not straightforward. In the field of headache disorders, although systematic classification and diagnostic criteria have been in place since 1988 [1], no definition of service quality existed until recently [2]. Lacking the grounding of any such definition, quality indicators proposed in the past have been partial and selective, and assessed for neither validity nor usability across headache-care settings [2].

The Global Campaign against Headache [3, 4], conducted by *Lifting the Burden* (LTB), a UK-registered non-governmental organization in official relations with the World Health Organization, aims inter alia to remedy deficiencies in headache-care worldwide. This requires a means of identifying such deficiencies. In a collaborative project with the European Headache Federation (EHF), LTB used various qualitative research methods to develop, through focus-group consultations and expert consensus, a definition of quality applicable to headache services.

“*Good-quality headache care achieves accurate diagnosis and individualized management, has appropriate referral pathways, educates patients about their headaches and their management, is convenient and comfortable, satisfies patients, is efficient and equitable, assesses outcomes and is safe*” [5].

Along with this definition, LTB proposed 30 indicators in nine quality domains that might be used to evaluate headache service quality, with the objectives not only identifying deficiencies but also of guiding their remedy. Each was associated with a specific assessment instrument.

The indicators were developed to be applicable across countries and cultures, and at all levels within health systems, in primary care and in specialist centres. No external standards were available to assess criterion-validity, which depended therefore on the methodological rigour employed in their development [5]. In a pilot implementation study conducted successfully in two specialist headache centres in Portugal and Germany [6], the assessment questionnaires were found easy to apply by health-care practitioners (HCPs) and patients, and not unduly time consuming [6]. Despite the differences in language and institutions (a university teaching hospital versus a private hospital), the findings were comparable in the two countries, suggesting operational validity in the evaluation of headache care. A subsequent pan-European study confirmed this in 14 specialist headache centres [7]. Further evaluations are nonetheless needed, and here we conduct a multicentre national study, extending implementation to the quality assessment of six Italian specialist headache centres (level three according to the EHF/LTB standard [8]) and examining acceptability and ease of use of the assessment instrument in another language and country.

## Methods

### Participating centres

Of more than 80 headache specialist centres in Italy, ten participate in a network (the Young Italian Headache Network) of young Italian researchers on headache; seven of these expressed their willingness to participate in the study. However, one centre discontinued its participation for unknown reasons, without contributing any data. The study was completed over 3 months, between September and December 2016, in the remaining six centres (Table 1). The centre of Roma Sapienza, involved also in the earlier study of Schramm et al. [7], collected new data in accordance with our study protocol.

**Table 1:** Characteristics of participating headache centres

	Place	Centre name	Level^a^	Service description	Participants (n)		
					Physicians	Other HCPs	Patients
1.	Bologna	Centro per lo Studio e la Cura delle Cefalee e delle Algie Facciali – Istituto delle Scienze Neurologiche di Bologna	3	Expert advice and care for patients with headache disorders and facial pain; headache research and teaching	3	6	60
2.	Firenze	Centro Cefalee e Farmacologia Clinica – AOUC Università degli studi di Firenze	3	University-based service and training centre run by headache experts; national referral centre for patients with refractory or rare headache disorders	4	2	60
3.	Modena	Centro Cefalee ed Abuso di Farmaci – Policlinico di Modena	3	University-based service run by headache-experienced pharmacologists supported by 2 nurses and one consultant psychiatrist	6	2	60
4.	Padova	Dipartimento delle Specialità Mediche – Neurologia – Centro Cefalee	3	Tertiary care centre for diagnosing and managing headache and other neurological disorders	2	3	60
5.	Roma Campus Bio-Medico	Centro per le Cefalee – Campus Bio-Medico di Roma	3	University-based neurological service provided by physicians experienced in headache disorders, cerebrovascular diseases and dementias	5	0	60
6.	Roma Sapienza	Centro di Riferimento Regionale per le Cefalee – Azienda Ospedaliera Sant’Andrea di Roma	3	University-based centre with daily outpatient, inpatient and ER service, supported by one psychologist and two nurses	5	3	60

^a^according to the EHF/LTB standard [8]

### Participants

In each centre we collected data from service records (via the service manager), from the physicians and other HCPs (nurses, psychologists and/or nursing assistants) involved in outpatient consultations, and from 60 consecutive patients (30 enrolled when making a first-time visit and 30 others undergoing a follow-up visit).

### Data collection, and instruments

We employed the five evaluation instruments used by Schramm et al. [7]. Table 2, set out similarly as in the earlier studies [6, 7], provides an overview of these. They included three different questionnaires, one each for the physicians, other HCPs and patients, all completed anonymously. The questionnaire for patients was formulated as an exit questionnaire, which they received from their HCPs at the end of their consultations and completed and returned before leaving. All questionnaires were translated from their English originals into Italian by two authors (LP and SG) according to *Lifting The Burden’s* translation protocol for hybrid documents [9], with TJS and physicians and patients not involved in the study providing review and assistance.

**Table 2:** Methods of implementation of service quality indicators

Indicator	Measure	Means of enquiry
Domain A. Accurate diagnosis is essential for optimal headache care		
A1. Patients are asked about the temporal profile of their headaches	a) Duration of presenting complaint is recorded in patient’s record (yes/no)	Patients’ records
A2. Diagnosis is according to current ICHD criteria	a) Diagnosis is recorded in patient’s record (yes/no)	Patients’ records
	b) Diagnostic record uses ICHD terminology (yes/no)	
A3. A working diagnosis is made at the first visit	Working diagnosis at first visit is recorded in patient’s record (yes/no)	Patients’ records
A4. A definitive diagnosis is made at first or subsequent visit	Definitive diagnosis is recorded in patient’s record or, if not, an appointment for review has been given (yes/no)	Patients’ records
A5. Diagnosis is reviewed during later follow-up	Diagnostic review during follow-up is routinely undertaken (yes/no)	Physicians’ questionnaire
A6. Diaries are used to support or confirm diagnosis	The service has a diagnostic diary available and physicians are aware of its availability (yes/no)	Physicians’ questionnaire
Domain B. Individualized management is essential for optimal headache care		
B1. Waiting-list times for appointments are related to urgency of need	a) Waiting-list times are recorded in database (yes/no)	Patients’ records
	b) A formal triage system exists to expedite appointments in cases of perceived urgency (yes/no)	Physicians’ questionnaire
B2. Sufficient time is allocated to each visit for the purpose of good management	a) Actual time (minutes) per visit is recorded by patient in exit questionnaire: 1st visits and follow-up visits	Patients’ questionnaire
	b) Patient is satisfied^a^ with actual time (yes/not yes)	Patients’ questionnaire
	c) Health-care providers express overall satisfaction (yes/no)	Physicians’ and other HCPs’ questionnaires
B3. Patients are asked about the temporal profile of their headaches	Frequency (or days/month) of symptoms is recorded in patient’s record (yes/no)	Patients’ records
B4. Treatment plans follow evidence-based guidelines, reflecting diagnosis	Prescribed drugs (names, doses and quantities) are recorded in patient’s record	Patients’ records
B5. Treatment plans include psychological approaches to therapy when appropriate	a) Access route to psychological therapies exists (yes/no)	Physicians’ questionnaire
	b) Utilisation is recorded in patient’s record	Patients’ records
B6. Treatment plans reflect disability assessment	a) An instrument for disability assessment is available(yes/no) and is appropriate in the setting (yes/no)	Physicians’ questionnaire
	b) Disability is recorded in patient’s record (yes/no)	Patients’ records
B7. Patients are followed up to ascertain optimal outcome	a) Follow-up appointment dates appear in central service records (yes/no)	Central service records
	b) A follow-up diary and/or calendar is available (yes/no)	Physician’s questionnaire
Domain C. Appropriate referral pathways are essential for optimal headache care		
C1. Referral pathway is available from primary to specialist care	A usable pathway exists (yes/no)	Physicians’ questionnaire
C2. Urgent referral pathway is available when necessary	A usable pathway exists (yes/no)	Physicians’ questionnaire
Domain D. Education of patients about their headaches and their management is essential for optimal headache care		
D1. Patients are given the information they need to understand their headache and its management	Patient is satisfied^a^ with information given (yes/not yes)	Patients’ questionnaire
D2. Patients are given appropriate reassurance	Patient is satisfied^a^ with information given (yes/not yes)	Patients’ questionnaire
Domain E. Convenience and comfort are part of optimal headache care		
E1. The service environment is clean and comfortable	a) Patient is satisfied^a^ with cleanliness and comfort (yes/not yes)	Patients’ questionnaire
	b) Health-care providers are satisfied with cleanliness and comfort (yes/no)	Physicians’ and other HCPs’ questionnaires
E2. The service is welcoming	Patient is satisfied^a^ with welcome (yes/not yes)	Patients’ questionnaire
E3. Waiting times in the clinic are acceptable	a) Waiting time (minutes) per visit is recorded by patient in exit questionnaire	Patients’ questionnaire
	b) Patient is satisfied^a^ with waiting time (yes/not yes)	Patients’ questionnaire
	c) Health-care providers are satisfied with waiting times (yes/no)	Physicians’ and other HCPs’ questionnaires
Domain F. Achieving patient satisfaction is part of optimal headache care		
F1. Patients are satisfied with their management	Patient is satisfied^a^ with overall management (yes/not yes)	Patients’ questionnaire
Domain G. Optimal headache care is efficient and equitable		
G1. Procedures are followed to ensure resources are not wasted	A protocol to limit wastage exists (yes/no)	Physicians’ questionnaire
G2. Costs of the service are measured as part of a cost-effectiveness policy	A record of input costs exists (yes/no)	Physicians’ questionnaire
G3. There is equal access to headache services for all who need it	A policy to ensure equal access exists (yes/no)	Physicians’ questionnaire
Domain H. Outcome assessment is essential in optimal headache care		
H1. Outcome measures are based on self-reported symptom burden (headache frequency, duration and intensity)	a) An outcome measure (HURT or similar) is available (yes/no)	Physicians’ questionnaire
	b) Outcomes according to this measure are recorded in patient’s record (yes/no/not applicable)	Patients’ records
H2. Outcome measures are based on self-reported disability burden	a) An outcome measure (HALT or similar) is available (yes/no)	Physicians’ questionnaire
	b) Outcomes according to this measure are recorded in patient’s record (yes/no/not applicable)	Patients’ records
H3. Outcome measures are based on self-reported quality of life	a) An outcome measure (WHOQoL or similar) is available (yes/no)	Physicians’ questionnaire
	b) Outcomes according to this measure are recorded in patient’s record (yes/no/not applicable)	Patients’ records
Domain I. Optimal headache care is safe		
I1. Patients are not over-treated^b^	Prescribed drugs (names, doses and quantities) are recorded in patient’s record (yes/no/not applicable)	Patients’ records
I2. Systems are in place to be aware of serious adverse events^C^	a) Serious adverse events are recorded	Patients’ records
	b) A protocol exists for reporting serious adverse events (yes/no)	Physicians’ questionnaire

*ICHD* International Classification of Headache Disorders, *HURT* Headache Under-Response to Treatment questionnaire [13, 14], *HALT* Headache-Attributed Lost Time questionnaire [11], *WHOQoL* World Health Organization Quality of Life questionnaire [12]

^a^Patients’ satisfaction was deduced from Likert scales, extending through the options (according to the question): “too little”, “about right”, “too much”; or “much too long”, “too long”, “reasonable”; or “very good”, “good”, “adequate”, “poor”, “very poor”. These were recoded as “yes” or “not yes” for analysis (“adequate” was coded as “yes”)

^b^Over-treatment may mean excessive use of drugs presumed able to induce medication overuse headache, overdosage with potentially harmful drugs such as ergotamine or steroids, use of prophylactic drugs for occasional headache or the wrong diagnosis, use of non-evidence-based treatments that are improbably effective

^c^Serious adverse events are those that cause death, are life-threatening, terminate or put at risk a pregnancy, or cause hospitalization, prolonged illness, disability and/or malignancy

In addition, data were extracted into two data-collection forms from the patients’ medical records and from service records. This was done by a local investigator at each centre who, to avoid any bias, did not perform any consultations with patients enrolled in the study.

### Data management and analysis

Data were entered into spreadsheets at each centre, and then transferred to the data collection centre (Headache and Drug Abuse Centre, University of Modena and Reggio Emilia).

Descriptive analysis was performed by LP. Demographic and clinical data were provided as numerical values and are summarized as percentages or mean values. No hyphotheses were statistically tested.

## Results

The characteristics of the study centres and participants (physicians, other HCPs and patients) are summarized in Table 1. We took data from a total of 360 patients and from two to six physicians and up to six other HCPs per centre. In one centre (Modena), one physician refused to complete the questionnaire; in all other cases, both HCPs and patients reported that the questionnaires were easy to apply, readily understood and not unduly time consuming. None of the indicators caused or led to difficulties on a practical level; the percentage of missing answers was zero.

Evaluation of each service according to the quality indicators is shown in Table 3, with results summarised below by domain.

**Table 3:** Outcomes of implementation of quality indicators by centre (% of positive answers)

	Bologna	Firenze	Modena	Padova	Roma Campus Bio-Medico	Roma La Sapienza
A1. Duration of complaint recorded	100	88	98	100	90	100
A2a. Diagnosis recorded	100	98	100	100	98	100
A2b. ICHD terminology used	100	98	100	100	97	100
A3. Working diagnosis at first visit recorded	100	98	100	100	98	100
A4. Definitive diagnosis or appointment for review	100	98	100	100	100	100
A5. Routinely diagnostic review during follow-up (physicians)	100	100	100	100	100	100
A6. Diagnostic diaries available (physicians)	100	75	100	100	100	100
B1a. Waiting-list times are recorded in database	0	0	0	0	0	0
B1b. Formal triage system exists (physicians)	100	75	100	100	100	100
B2a. Mean time per visit – 1st visits (minutes)	49	34	33	26	33	32
B2a. Mean time per visit – follow up visits (minutes)	29	17	28	22	29	20
B2b. Satisfaction with time per visit (patients)	97	90	87	95	97	92
B2c. Satisfaction with time per visit (physicians + HCPs)	100	83	100	100	100	100
B3. Frequency of symptoms is recorded	100	97	82	100	100	100
B4. Prescribed drugs (names, doses and quantities) are recorded	100	95	87	100	100	100
B5a. Access route to psychological therapies exists (physicians)	0	100	0	100	100	100
B5b. Utilisation of psychological therapies is recorded	n/a	n/a	n/a	n/a	100	100
B6a. Instrument for disability assessment is available (physicians)	0	75	80	100	0	0
B6b. Instrument for disability is appropriate in the setting (physicians)	n/a	75	80	100	n/a	n/a
B6c. Disability is recorded	0	0	0	0	0	0
B7a. Follow-up appointment dates appear in central service records	yes	yes	yes	yes	yes	yes
B7b. Follow-up diary/calendar is available (physicians)	100	100	100	100	100	100
C1. Referral pathway from primary care exists (physicians)	100	100	100	100	0	100
C2. Urgent referral pathway exists (physicians)	100	100	100	0	100	100
D1. Patient is satisfied with information given	97	90	85	93	95	97
D2. Patient is satisfied with reassurance given	85	85	82	87	87	95
E1a. Patient is satisfied with cleanliness and comfort	87	52	80	83	95	92
E1b. Physicians and HCPs are satisfied with cleanliness and comfort	67	50	100	100	100	100
E2. Patient is satisfied with welcome	83	68	88	90	98	88
E3a. Mean waiting time – 1st visits (minutes)	15	9	11	10	8	11
E3a. Mean waiting time – follow up visits (minutes)	15	9	20	5	5	25
E3b. Patient is satisfied with waiting times	70	73	70	73	82	68
E3c. Physicians and HCPs are satisfied with waiting times	11	50	57	80	80	100
F1. Patient is satisfied with overall management	83	80	78	90	95	90
G1. Protocol to limit wastage exists (physicians)	100	25	100	100	100	100
G2. Record of input costs exists (physicians)	100	50	100	0	100	100
G3. Policy to ensure equal access exists (physicians)	100	100	100	100	100	100
H1a. An outcome measure (HURT or similar) exists (physicians)	0	0	40	100	0	0
H1b. Outcomes are recorded	0	0	0	100	0	0
H2a. An outcome measure (HALT or similar) exists (physicians)	0	75	80	0	0	0
H2b. Outcomes are recorded	0	0	0	0	0	0
H3a. An outcome measure (WHOQoL or similar) exists (physicians)	0	0	0	0	0	0
H3b. Outcomes are recorded	0	0	0	0	0	0
I1. Prescribed drugs (names, doses and quantities) are recorded	100	95	87	100	100	100
I2a. Serious adverse events are recorded	0	0	0	0	0	0
I2b. Protocol for reporting serious adverse events exists (physicians)	100	75	100	100	100	100

*HCPs* Health-care providers, *ICHD* International Classification of Headache Disorders, *HURT* Headache Under-Response to Treatment questionnaire [13, 14], *HALT* Headache-Attributed Lost Time questionnaire [11], *WHOQoL* World Health Organization Quality of Life questionnaire [12]

### Domain A: accurate diagnosis

Diagnoses were recorded using ICHD terminology in ≥97% of cases. All diagnoses at every centre were documented after the first visit and reviewed during follow-up using diagnostic diaries. Temporal duration of the presenting headache disorder was reported in ≥88% of patients’ records (Table 3).

### Domain B: individualized management

Waiting-list times for first appointment were not documented, but all clinics reported a formal triage system to expedite appointments in case of perceived urgency.

Mean time allocated to patients’ visits ranged, according to patients’ reports, between 26 (Padova) and 49 min (Bologna) for the first visit and between 17 (Firenze) and 29 min (Bologna and Roma Campus Bio-Medico) for follow-up visits. Patients’ and HCPs’ satisfaction with time per visit was ≥83% in all centres.

Frequency of symptoms and treatment plans were properly reported in most cases (respectively >82 and >87%). Psychological therapies were available in four of the six centres: in two of these (Roma Campus Bio-Medico and Roma Sapienza) their prescription was correctly reported in patients’ records, while in the other two (Padova and Firenze) these therapies were not prescribed to any patients enrolled in the study. Disability instruments were available in three centres (Padova, Modena and Firenze), but no HCP utilized them regularly. Follow-up calendars were available and recorded future appointment dates in all centres (Table 3).

### Domain C: appropriate referral pathways

Referral pathways were well established in most centres: exceptions were Roma Campus Bio-Medico for primary care and Padova for urgent consultations (Table 3).

### Domain D: education and reassurance of patients

Most patients (> 85%) expressed satisfaction with the information given by physicians, and >82% declared they had received appropriate reassurance (Table 3).

### Domain E: convenience and comfort

Both patients and HCPs found the service environment clean and comfortable in four centres, with some dissatisfaction expressed in two (Bologna and Firenze). Patients’ satisfaction was generally high with the welcome they received from the services (67–98%) and with waiting times to be seen in the centres (67–82%). Average waiting times varied little, 8–15 min for first visits and 5–25 min for follow-up visits, and were unsatisfactory only for the HCPs in one centre (Bologna) (Table 3).

### Domain F: patient satisfaction

Overall satisfaction with their management was expressed by 78–95% of patients. (Table 3).

### Domain G: efficiency and equitability

All clinics had a protocol to avoid wastage of resources, and input costs were regularly recorded, but not all physicians were aware of these in one centre (Firenze) while the information was available to senior management only in another (Padova). All centres provided equal access for all who might need it.

### Domain H: outcome assessment

Outcome assessments were made routinely only in Padova, and were based on symptom burden. Neither disability nor quality of life were assessed routinely at any centre.

### Domain I: safety

All services appeared safe: prescribed drugs were well documented, protocols for reporting serious adverse events (SAEs) existed in each centre and there were no recorded SAEs.

## Discussion

The study was planned as a multicentre national service quality evaluation. It was conducted in six Italian specialist headache centres, and has shown common practices among them. The centres were all university teaching hospitals, with similar settings and levels of care, and operating within a common culture, with one language and in the same health service. The Italian National Health Service (NHS) has some differences between the administrative regions of Italy, but everywhere provides citizens with free access to a general practitioner, a general paediatrician and admission without charge to hospitals. Specialist consultations, as for headache, are provided with a small charge. Specifically in palliative care, Italian law establishes citizens’ right of access to pain care centres for both oncological and non-oncological pain.

However, our objective was not to compare the service quality of these centres but to establish, in Italian headache care, the usability, practicality and acceptability of the quality indicators, and their associated assessment instruments, developed by LTB. The six centres all collected data efficiently, found the questionnaires to be readily understood and easily applied, and, importantly, the whole process was not unduly time consuming. These findings accorded with those of the previous studies with similar purpose [6, 7]. Consequently, we believe the indicators can be used by the Italian headache scientific community to identify majority practices while simultaneously recognizing deficiencies, and therefore to set benchmarks against which quality may be judged in future in this country.

We should nonetheless comment on the specific findings. The results were mostly positive in all 30 quality indicators, but a few deficits were uncovered. In domain A, diagnoses utilized current ICHD criteria, and were routinely reviewed during follow-up; diagnostic diaries were regularly used in each centre. The influence of the continuous efforts of Italian scientific societies to facilitate application of the ICHD criteria is gratifyingly in evidence here. In domain B, triage systems were in place to adjust waiting times to urgency of need, an important consideration. Most patients and physicians were satisfied with the time given in consultations, and with waiting times. Follow-up calendars were available in each centre. On the other hand, no centre recorded in service records the average waiting times for a visit, while HCPs perceived that times were too long for high-quality care, a consequence of the high numbers of patients seeing the relatively few doctors working in each centre. Psychological therapies were provided by four centres, with different modalities, a disparity probably due to the difficulties in recruiting psychologists in the NHS (often they are private specialists). Surprisingly, despite that headache disorders are collectively the third highest cause of years lived with disability (YLDs) worldwide [10], no centre recorded patients’ disability scores. In domain C, referral pathways were in place in five of six centres. Importantly, most patients were generally satisfied and felt welcomed, informed and reassured (domains D, E and F). Furthermore, in domain G, every centre achieved well in the use of protocols to avoid wastage of resources and in promoting equitable access, while in domain I, every service appeared safe.

A critical issue emerged in the lack of formal outcome assessment (domain H), indicating the need for greater use of, above all, disability and quality of life instruments (HALT [11] and WHOQoL [12], or similar). In two services (Firenze and Modena), even though the physicians were well aware of the availability of validated and easy-to-apply instruments, and of their importance, nobody used them during follow-up because of time constraints. All centres complained that their use was not practicable because of lack of time for HCPs to administer them. Nonetheless, without formal and standardized outcomes assessment, it was unclear how the centres established the effectiveness of their interventions against headache. One centre (Padova) routinely used a symptom measure similar to HURT [13, 14], and all others employed some measure of the frequency and severity of headache, such as a headache index and/or numerical rating scales.

### Potential for benchmarking

The consensus group developing these indicators foresaw their employment in benchmarking, but deferred exploration of this potential important application [5]. Our findings in these six centres suggest that the indicators may indeed be deployed in setting benchmarks – at least in Italian specialist headache care. They clearly identified majority practice, with some aspects requiring improvement. Majority practice fails to make routine use of formal outcome measures, despite that this is an obvious part good record-keeping, and would appear to be necessary to judge whether best outcomes are achieved. The evident practical (lack-of-time) difficulty in doing this needs to be addressed. It should be recognized that HURT, for example, is intended for self-completion [13, 14], and can be brought by patients to follow-up visits already filled in – and not only used for its purpose of guiding treatment (less necessary in specialist care) but also placed in the patients’ records. The solution may lie in developing an electronic version, which has not so far been done. In the context of benchmarking, there are aspects of care not currently included in the quality indicators that many might think should be, and which at least deserve to be discussed for the future. One that is clearly relevant is whether diagnostic examinations and the prescriptions of treatments follow evidence-based guidelines [15, 16]. The consensus group responsible for developing the indicators acknowledged this, but also recognized that assessment of these was highly problematic [5]. A second aspect, not included in the validated set of quality indicators but perhaps important nonetheless, is the non-medical barriers to care erected, before visits, by various impositions on patients (finding and paying for parking space, for example). We asked patients about their satisfaction with regard to this particular aspect, in a similar manner to the other quality indicators (“yes”/“not yes” responses), obtaining positive replies as follows: Firenze 45%, Roma Sapienza 53%, Modena 68%, Padova 80%, Bologna 82%, Roma Campus Bio-Medico 87%. Although not a validated quality indicator, this (or an adaption of it) might identify additional difficulties encountered by patients in receiving medical care from the specialist centres that are relevant to service quality.

## Conclusions

This Italy-wide survey, the first such study to examine headache service quality indicators, showed in six specialist centres that the indicators are fit for purpose, identifying many commonalities and detecting deficits as a guide to quality improvement.

While it questioned more than 40 health-care workers and 360 patients, it was deliberately restricted to specialist services operating in similar settings. There was purpose in this, since it was the indicators themselves, and their application, that were being assessed. On the other hand, it was a limitation. Among the next steps is to expand their utilization into more varied primary- and secondary-care settings; most headache patients look for treatment in non-specialist care [16]. Although quality may be an absolute attribute, it is not necessarily attainable to perfection, or there may be practical or cost constraints that plead for compromise. Accordingly, benchmarks may be different in primary care, and this is a pressing area of enquiry. We expect that the quality indicators can be applied in primary care, perhaps with some refinement, but this is yet to be demonstrated.
